# Diagnostic Performance of Cardiac Computed Tomography for Detecting Patent Foramen Ovale: Evaluation Using Transesophageal Echocardiography and Catheterization as Reference Standards

**DOI:** 10.3390/jcdd10050193

**Published:** 2023-04-26

**Authors:** Takashi Miki, Koji Nakagawa, Keishi Ichikawa, Tomofumi Mizuno, Rie Nakayama, Kentaro Ejiri, Satoshi Kawada, Yoichi Takaya, Masakazu Miyamoto, Toru Miyoshi, Teiji Akagi, Hiroshi Ito

**Affiliations:** 1 Department of Cardiovascular Medicine, Okayama University Graduate School of Medicine, Dentistry and Pharmaceutical Sciences, Okayama 700-8558, Japan; 2 Department of General Internal Medicine 3, Kawasaki Medical School, Okayama 700-8505, Japan

**Keywords:** patent foramen ovale, cardiac computed tomography, transesophageal echocardiography, catheterization, channel-like appearance, channel-like appearance with contrast jet flow

## Abstract

Background: Patent foramen ovale (PFO) is associated with various diseases such as cryptogenic stroke, migraine, and platypnea–orthodeoxia syndrome. This study aimed to evaluate the diagnostic performance of cardiac computed tomography (CT) for PFO detection. Materials and Methods: Consecutive patients diagnosed with atrial fibrillation and who underwent catheter ablation with pre-procedural cardiac CT and transesophageal echocardiography (TEE) were enrolled in this study. The presence of PFO was defined as (1) the confirmation of PFO using TEE and/or (2) the catheter crossing the interatrial septum (IAS) into the left atrium during ablation. CT findings indicative of PFO included (1) the presence of a channel-like appearance (CLA) on the IAS and (2) a CLA with a contrast jet flow from the left atrium to the right atrium. The diagnostic performance of both a CLA alone and a CLA with a jet flow was evaluated for PFO detection. Results: Altogether, 151 patients were analyzed in the study (mean age, 68 years; men, 62%). Twenty-nine patients (19%) had PFO confirmed by TEE and/or catheterization. The diagnostic performance of a CLA alone was as follows: sensitivity, 72.4%; specificity, 79.5%; positive predictive value (PPV), 45.7%; negative predictive value (NPV), 92.4%. The diagnostic performance of a CLA with a jet flow was as follows: sensitivity, 65.5%; specificity, 98.4%; PPV, 90.5%; NPV, 92.3%. The diagnostic performance of a CLA with a jet flow was statistically superior to that of a CLA alone (*p* = 0.045), and the C-statistics were 0.76 and 0.82, respectively. Conclusion: A CLA with a contrast jet flow in cardiac CT has a high PPV for PFO detection, and its diagnostic performance is superior to that of a CLA alone.

## 1. Introduction

Patent foramen ovale (PFO) is a persistent connection between the left and right atria that is a result of the incomplete fusion of the septum primum and the septum secundum. PFO is observed in approximately 25% of the general population [1] and is associated with various diseases such as cryptogenic stroke, migraine, and platypnea–orthodeoxia syndrome [2,3,4,5]. In symptomatic patients with PFO, transcatheter PFO closure is the treatment choice. Several recent trials have demonstrated that device closure for PFO was superior to medical therapy with respect to stroke recurrence and provided long-term benefits in terms of oxygen saturation for patients with PFO-related hypoxemia [6,7,8,9]; therefore, the importance of PFO diagnosis has been increasing. Transesophageal echocardiography (TEE), including the microbubble test, is the gold standard for the diagnosis of PFO [10,11]. However, TEE is a semi-invasive method, and severe complications, such as esophageal perforation, have been reported [12,13]. Moreover, TEE is not always tolerated well in patients who have a strong pharyngeal reflex.

Advances in multidetector computed tomography (CT) technology have allowed the precise imaging of the cardiovascular system, including that of not only the coronary arteries but also other cardiac structures. Electrocardiography (ECG)-gated cardiac CT allows the identification of the morphology of the interatrial septum (IAS) [14]. Some previous studies have described the diagnostic value of cardiac CT [15,16], but the diagnosis of PFO using CT is not well established in current clinical practice. However, all these studies evaluated the presence of PFO with TEE as a reference standard. The diagnostic sensitivity and specificity of TEE with respect to PFO was approximately 89% and 91%, respectively [17]; thus, some cases in these previous studies showed a failure to diagnose PFO with TEE alone, and, in others, PFO appeared to be present in the TEE findings but was actually absent. Cardiac catheterization, although invasive, is another diagnostic method for PFO. Regardless of TEE findings, the direct crossing of the catheter from the right atrium to the left atrium via the IAS is a confirmation for the presence of an interatrial shunt. At our institution, cardiac CT and TEE before catheter ablation for atrial fibrillation (AF) are performed in principle. Moreover, crossing the catheter into the IAS is conducted in most cases. This study aimed to evaluate the diagnostic performance of ECG-gated cardiac CT for the identification of PFO in comparison to TEE and/or catheter crossing into the IAS as reference standards.

## 2. Materials and Methods

### 2.1. Study Population

**Figure 1:**
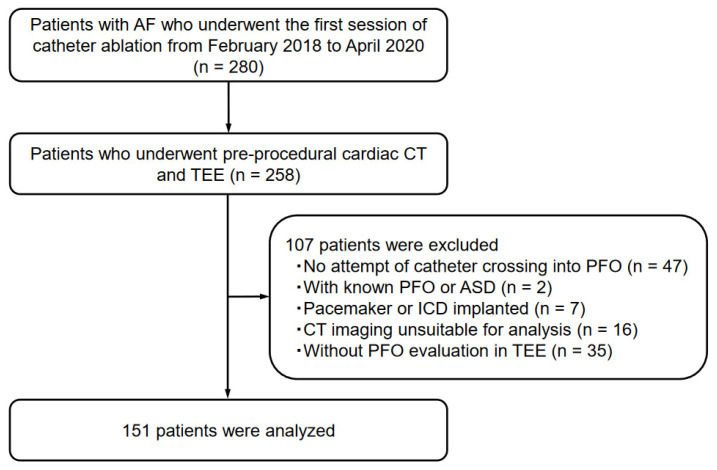
Patient enrollment in the present study.

This study included consecutive patients diagnosed with AF who underwent the first session of catheter ablation at Okayama University Hospital between February 2018 and April 2020 (*n* = 280). From the study population, 258 patients who underwent both pre-procedural cardiac CT and TEE, followed by catheter ablation, were included. Forty-seven patients with no attempt of catheter crossing into the IAS, two patients with known PFO or atrial septal defect, seven patients with cardiac implantable electronic devices, sixteen patients with suboptimal CT imaging acquisition or without ≤1 mm thickness of CT imaging, and thirty-five patients who had not undergone TEE for the evaluation of PFO were excluded. Therefore, 151 patients were analyzed as the study population (Figure 1). Patients’ clinical data, such as comorbidities and transthoracic echocardiographic (TTE) findings, were collected from the medical records. 

The following abbreviations are defined as follows: AF, atrial fibrillation; CT, computed tomography; TEE, transesophageal echocardiography; PFO, patent foramen ovale; ASD, atrial septal defect; ICD, implantable cardioverter defibrillator.

This study was approved by the Ethics Committee of Okayama University Graduate School of Medicine, Dentistry, and Pharmaceutical Sciences (Okayama, Japan), and the requirement to obtain informed consent was waived. The study was conducted in accordance with the principles of the Declaration of Helsinki.

### 2.2. CT Image Acquisition

All CT scans were performed on a 128-slice CT scanner (SOMATOM Definition Flash; Siemens Medical Solutions, Erlangen, Germany). Cardiac CT images were obtained using the following parameters: detector collimation, 64 × 0.6 mm—equaling a slice acquisition of 128 × 0.6 mm; table pitch adapted to heart rate, 0.17–0.38; rotation time, 275 ms; tube current time product, 360 mAs; tube voltage, 120 kV. All patients arrived at the hospital an hour before the scheduled CT scanning time, and those with a heart rate of ≥60 beats/min received an oral β-blocker. If the heart rate did not decrease to <60 beats/min, the patient was administered an intravenous β-blocker to further reduce the heart rate. The initial bolus of contrast agent (Omnipaque 350, Daiichi Sankyo, Tokyo, Japan) was calculated as body weight × 0.07 mL and injected over 10 s. A test bolus CT acquisition protocol was adopted and performed at the level of the ascending aorta after the administration of 10 mL of contrast medium, followed by a second bolus consisting of 80% of the initial volume of contrast medium diluted to 50% with normal saline and then a compensatory 20% bolus of normal saline. Axial images were obtained at 70% of the R–R interval for each cardiac cycle using a section thickness of 0.75 mm. If an AF rhythm was present at the time of CT scan, CT images were obtained using the retrospective ECG-gating method and reconstructed at the best phase with fewer artifacts.

### 2.3. Assessment of PFO at Cardiac CT

Double-oblique images perpendicular to the atrial septum were reconstructed at a workstation (SYNAPSE VINCENT, Fujifilm Medical, Tokyo, Japan). Image analysis was performed by two independent trained cardiologists (T.M. and K.I.). CT analysts analyzing cardiac CT were blinded to the other patient data such as TEE.

**Figure 2:**
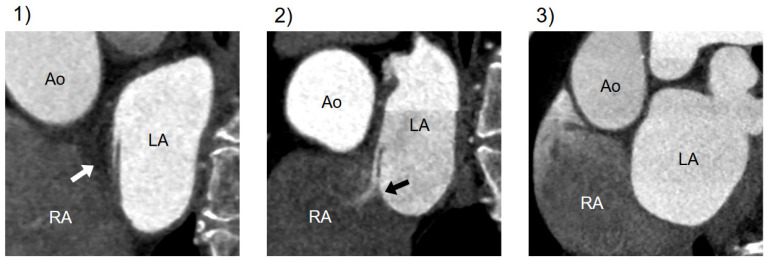
Variations in CT imaging on the atrial septum. Cardiac CT findings of the atrial septum on a short-axis view through the atria: (**1**) CLA without contrast jet flow; (**2**) CLA with jet flow toward RA; (**3**) normal interatrial septum without a visible channel. CT, computed tomography; Ao, aorta; LA, left atrium; RA, right atrium; CLA, channel-like appearance.

CT findings related to PFO were categorized into three types: (1) presence of channel-like appearance (CLA), (2) CLA with jet flow of the contrast media toward the right atrium, and (3) absence of a visible channel [15] (Figure 2).

### 2.4. PFO Confirmation

The presence of PFO was defined as (1) confirmation of PFO using TEE or (2) catheter crossing the IAS into the left atrium. TEE was performed on iE33 using the X7-2t probe (Philips Medical System) under local anesthesia. A microbubble test was performed to confirm the right-to-left shunt via PFO at rest and under spontaneous Valsalva maneuver several times. The PFO using TEE was confirmed by the presence of a separation between the septum primum and the septum secundum or by microbubbles crossing from the right atrium into the left atrium through the IAS.

Crossing of catheters into the IAS was attempted in all participants before septal puncture during the AF ablation procedure. A decapolar deflectable electrode catheter (EPstar Snake; Japan Lifeline, Japan, Tokyo) was used for PFO crossing under the guidance of fluoroscopy and intracardiac echocardiography.

### 2.5. Risk Factors for Cardiovascular Disease, Laboratory Analyses, and TTE Data

Information on demographics, smoking status, medication, and blood samples was collected for each patient before catheter ablation. Smoking was defined as current cigarette smoking. Obesity was defined as a body mass index of >25 kg/m^2^. Hypertension was defined as a seated blood pressure of ≥140/90 mmHg or the use of antihypertensive agents. Diabetes mellitus was defined as undergoing antidiabetic treatment or according to the criteria set by the American Diabetes Association [18]. Dyslipidemia was defined as the use of cholesterol-lowering medications or having one or more of the following conditions: (1) serum triglyceride level of ≥150 mg/dL, (2) high-density lipoprotein cholesterol level of <40 mg/dL, and (3) low-density lipoprotein cholesterol level of ≥140 mg/dL. Laboratory parameters were measured using an automated analyzer using standard laboratory techniques. TTE was performed to assess the left ventricular ejection fraction (LVEF) calculated according to the biplane method of disk summation, the left atrium volume index (LAVI), and E/e′. 

### 2.6. Statistical Analysis

Continuous variables are presented as mean ± standard deviation or median (interquartile range). Categorical variables are presented as frequency and proportion (%). Differences between the two groups were evaluated using χ^2^ tests for categorical variables and Student’s *t*-tests or Mann–Whitney *U* tests for continuous variables, as appropriate. The sensitivity, specificity, positive predictive value (PPV), negative predictive value (NPV), and accuracy of cardiac CT for the detection of PFO according to the different PFO appearance types (presence of CLA alone or CLA with a jet flow) were calculated. The receiver operating characteristic (ROC) curve analyses for the two types of CT appearance were performed, and the DeLong test was used to compare the C-statistics. A *p*-value of <0.05 was considered statistically significant. All statistical analyses were performed using SPSS 24.0 for Windows (IBM Corp., Armonk, NY, USA) and R statistical package (version 3.5.2; R Foundation for Statistical Computing, Vienna, Austria).

## 3. Results

### 3.1. Patient Characteristics

The baseline patient characteristics are summarized in Table 1. The mean age of the participants was 68 ± 9 years, and 62% of the participants were men. Moreover, 62% of patients had paroxysmal AF, and the remaining patients had persistent AF. Approximately 8% of patients had a history of ischemic stroke, and the mean CHADS_2_ score was 1.4 ± 1.0. In terms of echocardiographic parameters, the mean LVEF was 62.2% ± 7.8% and the LAVI was 44.7 ± 13.8 mL/m^2^. A comparison of the baseline characteristics of patients with and without PFO as confirmed by TEE or catheter crossing showed no significant difference in any variables. 

### 3.2. PFO Diagnosis with TEE and Catheterization

PFO was diagnosed in 21 (14%) of the 151 patients using TEE and in 23 patients (15%) using catheter crossing into the IAS toward the left atrium. As a result, 29 (19%) of the 151 patients were confirmed to have PFO using either TEE or catheterization.

### 3.3. PFO Detection and Diagnostic Performance at CT Imaging

On cardiac CT images, a CLA of the IAS was observed in 46 (30%) of the 151 patients; meanwhile, a CLA with a contrast jet flow was detected in 21 patients (14%).

The diagnostic performance of cardiac CT concerning PFO detection is shown in Table 2. The diagnostic performance of a CLA alone was as follows: sensitivity, 72.4%; specificity, 79.5%; PPV, 45.7%; NPV, 92.4%; accuracy, 78.1%. The diagnostic performance of a CLA with a jet flow was as follows: sensitivity, 65.5%; specificity, 98.4%; PPV, 90.5%; NPV, 92.3%; accuracy, 92.1%.

The median dose-length product for the acquisition of cardiac CT images was 1745.8 mGy·cm (interquartile range, 1265.1–2194.2 mGy·cm).

### 3.4. Comparison of Diagnostic Performance of CT Findings: CLA Alone versus CLA with Jet Flow

**Figure 3:**
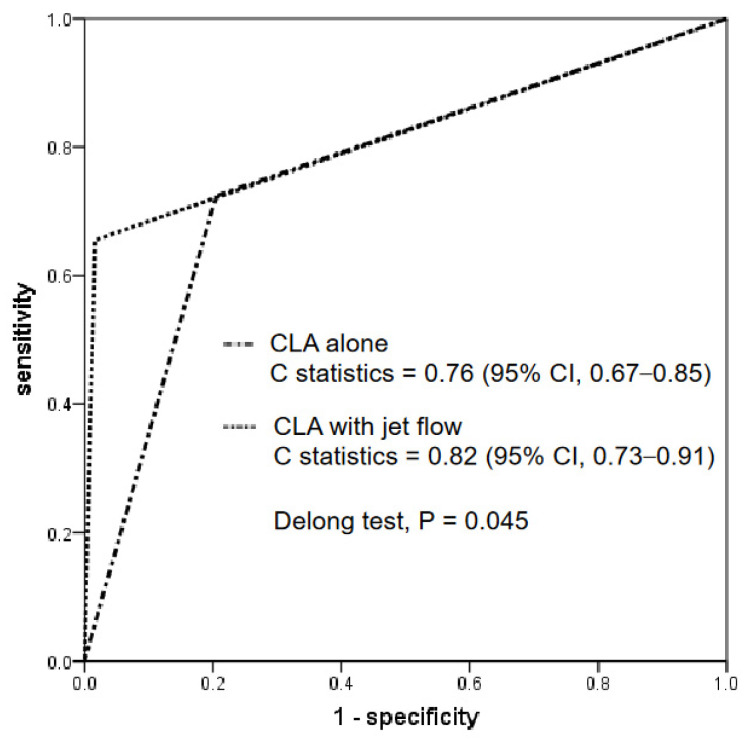
ROC curve analysis. The diagnostic performances between a CLA with and without a jet flow are compared. The C-statistics of a CLA with a jet flow were significantly greater than those of CLA alone. ROC, receiver operating characteristic; CLA, channel-like appearance.

Figure 3 shows the ROC curve analysis of the CT findings for a CLA alone and a CLA with a contrast jet flow. The C-statistics for each were 0.76 (95% CI, 0.67–0.85) and 0.82 (95% CI, 0.73–0.91), respectively. The DeLong test revealed that the C-statistics of a CLA with a jet flow were significantly greater than those of a CLA alone (*p* = 0.045). 

### 3.5. Effect of AF during CT Acquisition for PFO Diagnosis

Of 151 patients, 60 (40%) showed the presence of an AF rhythm at the time of CT. To evaluate the effect of AF on CT diagnostic performance for PFO detection, the detection rate of CLA with a contrast jet flow into the right atrium and the diagnostic accuracy of CLA with a jet flow were compared between patients with and without AF during CT acquisition. CLA with a contrast jet flow was detected in 11 of 60 patients with AF rhythm at CT scanning (18.3%) and in 10 of 91 patients without AF (11.0%), and there was no significant difference between the two groups (*p* = 0.202). The diagnostic accuracy for the group with AF and the group without AF was 97% and 90%, respectively (*p* = 0.277); therefore, AF rhythm at CT acquisition was not associated with the diagnostic performance of cardiac CT for the detection of PFO.

## 4. Discussion

To the best of our knowledge, this is the first study to demonstrate the diagnostic performance of cardiac CT for the detection of PFO using TEE and/or catheter crossing into the IAS as reference standards. The major findings of the present study were as follows: (1) the diagnostic performance of cardiac CT was clinically feasible for the detection of PFO; (2) a CLA with a contrast jet flow on cardiac CT increased the specificity and PPV for the detection of PFO and showed a statistically better diagnostic performance than a CLA alone.

Although TEE is the gold standard for the evaluation of PFO [19], it sometimes fails to diagnose PFO, and its sensitivity has been reported to be approximately 89% [17,20]. Some PFOs are diagnosed using catheterization even in patients in whom TEE fails to detect PFO [21]. PFO is reported in 20%–25% of the population [1], and in the present study, we were able to obtain a diagnosis rate of PFO of 19%, which was similar to the general prevalence using TEE and catheter crossing into the IAS as reference standards for PFO diagnosis. In several studies, the diagnostic performance of cardiac CT for the detection of PFO was validated using TEE as a gold standard [15,16,22], but no previous study has reported the use of TEE and catheterization as standards.

In previous studies, a CLA of the IAS and a CLA with a visible jet flow of contrast media in cardiac CT have been described as findings indicative of PFO [15,16]. Kim et al. reported that CLA with a jet flow had a higher PPV for PFO diagnosis (90.5%) than a CLA alone (52.6%) [15]. The present study also demonstrated that a CLA with a jet flow had a high PPV (90.5%), and the diagnostic performance of a CLA with a jet flow was statistically superior to a CLA alone by C-statistics. Therefore, a CLA with a jet flow of a contrast agent toward the right atrium in cardiac CT images is strongly indicative of the presence of PFO; however, a CLA without a jet flow did not reliably confirm PFO. A CLA without a jet flow in CT findings seems to represent the following cases: (1) although PFO is actually present, a contrast agent flow through the PFO is not detected at CT acquisition; (2) there is an atrial septal pouch, which is the incomplete fusion of the septal primum and the septal secundum. The septal pouch was reported to be observed in 60% of autopsied hearts without PFO, which is not a rare interatrial structure [23].

Cardiac CT is used to detect a left-to-right shunt via PFO. Some previous studies using TEE to diagnose PFO have shown that left-to-right shunts were common in the majority of PFO patients [24,25]. Left atrial pressure is generally higher than right atrial pressure throughout most of the cardiac cycle, which would more likely result in a PFO-mediated left-to-right shunt.

Because this study was performed in patients with AF who were scheduled to undergo catheter ablation, 40% of patients showed the presence of an AF rhythm at CT acquisition. Nevertheless, the detection rate of a CLA with a contrast jet flow and the diagnostic accuracy of PFO did not differ between patients with and without AF at CT acquisition. A previous study has indicated that a contrast jet flow through the PFO was mostly detected between 60% and 70% of the R–R interval [16], and cardiac CT images in the present study were acquired at similar phases in many subjects. The 60%–70% interval of the cardiac cycle is usually the previous phase of the P wave on ECG; therefore, an atrial contraction could not be a crucial factor for detecting a contrast jet flow via PFO. It is, therefore, assumed that an AF rhythm at CT acquisition did not affect PFO diagnosis with cardiac CT.

In the present study, the diagnostic sensitivity of a CLA with a jet flow was 65.5%, suggesting that TEE is superior in ruling out PFO in patients suspected of cryptogenic stroke. However, a recent report demonstrated that retrospective ECG-gated cardiac CT throughout the full cardiac cycle can improve the detectability of PFO compared to single-phase cardiac CT [16]. If further studies establish that some imaging acquisition methods increase the diagnostic sensitivity of cardiac CT for the detection of PFO, cardiac CT may be used as an alternative method to TEE for the detection of PFO, particularly in patients with stroke who have difficulty undergoing TEE.

The present study had some limitations. First, this was a single-center study, and the study population was relatively small. Second, there was selection bias because the study did not include patients with cryptogenic stroke via PFO. Therefore, in these patients, the diagnostic performance of CT for the detection of PFO may be different from the overall results of this study. Finally, PFO may have gone undetected, even if the PFOs in this study were confirmed using TEE and catheterization.

## 5. Conclusions

The diagnostic performance of cardiac CT for the detection of PFO is clinically feasible. Our study demonstrated that the left-to-right contrast flow through the CLA of the IAS using cardiac CT had a high PPV for PFO detection and indicated a better diagnostic performance than the CLA alone, with the use of TEE and catheterization as reference standards. Our findings suggest that a CLA with a jet flow is strongly indicative of the presence of PFO in routine cardiac CT acquisition.

## Figures and Tables

**Table 1:** Comparison of baseline characteristics between patients with and without PFO.

	All (*n* = 151)	PFO Present (*n* = 29)	PFO Absent (*n* = 122)	*p*-Value *
Age, years	68 ± 9	70 ± 6	68 ± 10	0.298
Male sex	94 (62)	19 (66)	75 (62)	0.687
Hypertension	93 (62)	17 (59)	76 (62)	0.715
Dyslipidemia	48 (32)	10 (35)	38 (31)	0.729
Diabetes mellitus	27 (18)	7 (24)	20 (16)	0.328
Smoking	14 (9)	2 (7)	12 (10)	0.624
Paroxysmal AF	94 (62)	14 (48)	80 (66)	0.084
AF at CT acquisition	60 (40)	14 (48)	46 (38)	0.296
History of ischemic stroke	12 (8)	3 (10)	9 (7)	0.595
Obesity	62 (41)	8 (28)	54 (44)	0.101
CHADS_2_ score	1.4 ± 1.0	1.6 ± 1.1	1.3 ± 1.0	0.241
BNP, pg/mL	113 [57–229]	156 [50–361]	105 [57–213]	0.289
LVEF, %	62.2 ± 7.8	59.5 ± 9.2	62.9 ± 7.3	0.074
LA volume index, mL/m^2^	44.7 ± 13.8	45.4 ± 11.5	44.5 ± 14.3	0.743
E/e′	13.0 ± 7.3	12.9 ± 5.2	13.0 ± 7.7	0.954

Data are presented as mean ± standard deviation, number (%), or median [interquartile range], as appropriate. PFO, patent foramen ovale; AF, atrial fibrillation; CT, computed tomography; BNP, brain-type natriuretic peptide; LVEF, left ventricular ejection fraction; LA, left atrium. * Comparison between PFO present and absent groups.

**Table 2:** Diagnosis of PFO at cardiac CT findings.

	Sensitivity, %	Specificity, %	PPV, %	NPV, %	Accuracy, %
CLA alone	72.4	79.5	45.7	92.4	78.1
CLA with jet flow	65.5	98.4	90.5	92.3	92.1

PFO, patent foramen ovale; PPV, positive predictive value; NPV, negative predictive value; CLA, channel-like appearance.

## Data Availability

The data presented in this study are available on request from the corresponding author. The data are not publicly available due to privacy reasons.

## List of Abbreviations

PFO	patent foramen ovale
TEE	transesophageal echocardiography
IAS	interatrial septum
AF	atrial fibrillation
CLA	channel-like appearance
